# Retraction: Upregulation of HYAL1 Expression in Breast Cancer Promoted Tumor Cell Proliferation, Migration, Invasion and Angiogenesis

**DOI:** 10.1371/journal.pone.0277500

**Published:** 2022-11-07

**Authors:** The PLOS ONE Editors

After this article [1] was published, concerns were raised about the mouse tumor sizes reported in Fig 7. Specifically, the chart in Fig 7A of the article appears to report tumor sizes of up to 3500 mm^3^.

In response to queries about these experiments, the first author stated that when this study was conducted, the animal monitoring procedures established by the institution were followed. They stated that the appearance (skin, hair, limbs, tail, eyes, nostrils, ears, mouth), behavior, mental condition, eating, drinking, urine and feces of the experimental animals were observed and recorded daily. The first author also stated that when the pain and distress of the experimental animals was ‘more than expected’, or at the end of the experiment, the experimental animals were euthanized using ether as an inhalation anesthetic, and cervical spine dislocation. The authors did not provide details as to how pain and distress were evaluated, what specific threshold was applied as an endpoint criterion, or whether any animals died or were euthanized before the 9-week endpoint.

The first author provided the underlying individual-level tumor volume and tumor weight data. This showed that the individual mouse tumor sizes were up to 4621.72 mm^3^ and many individual mouse tumor sizes were over 2000 mm^3^, particularly in the MCF-HYAL1 group from 7 weeks. The large tumors (especially those in the 2000–4622 mm^3^ range) would be expected to impede mobility and cause significant pain and discomfort. Furthermore, the quantitative results reported in Fig 7A call into question whether the 9-week endpoint and large tumor sizes were scientifically justified: the figure reports statistically significant differences between groups as early as week 5, when tumors were < 2000 mm^3^ in volume.

Based on our assessment and advice received previously from a laboratory animal welfare expert, *PLOS ONE* concluded that the reported tumor sizes, study design, and endpoint criteria would likely not have met internationally accepted animal research ethics standards that were in place when the study was conducted.

*PLOS ONE* is also concerned about the use of ether as an anesthetic agent since this agent can cause irritation and distress for laboratory animals and also presents risks to laboratory personnel.

The authors stated that the ethics approval letter for this study is no longer available.

In light of the above concerns, *PLOS ONE* concluded that the study did not comply with the journal’s Animal Research Policy which requires that studies involving animals must have been conducted according to internationally accepted standards. Therefore, *PLOS ONE* retracts this article. The editors regret that these concerns were not addressed prior to the article’s publication.

JXT did not respond to the retraction decision. XYW, XLS, HYL, YS, LW and GSR did not respond or could not be reached.

## References

[1] Tan J-X, Wang X-Y, Su X-L, Li H-Y, Shi Y, et al. (2011) Upregulation of HYAL1 Expression in Breast Cancer Promoted Tumor Cell Proliferation, Migration, Invasion and Angiogenesis. PLoS ONE 6(7): e22836. 10.1371/journal.pone.0022836 21829529 PMC3145763

